# Clinical outcomes between calcium channel blockers and angiotensin receptor blockers in hypertensive patients without established cardiovascular diseases during a 3-year follow-up

**DOI:** 10.1038/s41598-021-81373-7

**Published:** 2021-01-19

**Authors:** Han Saem Jeong, Hong‐Seok Lim, Hun-Jun Park, Wang-Soo Lee, Jin-Oh Choi, Hui Seung Lee, Sang-Ho Jo, Soon Jun Hong

**Affiliations:** 1 Dr.Jeong’s Heart Clinic, Jeonju, Republic of Korea; 2 Department of Cardiology, Ajou University School of Medicine, Suwon, Republic of Korea; 3 Division of Cardiology, Department of Internal Medicine, Seoul St. Mary’s Hospital, The Catholic University of Korea, Seoul, Republic of Korea; 4 Division of Cardiology, Department of Internal Medicine, College of Medicine, Chung-Ang University Hospital, Seoul, Republic of Korea; 5 Division of Cardiology, Department of Medicine, Heart Vascular Stroke Institute, Samsung Medical Center, Sungkyunkwan University School of Medicine, Seoul, Republic of Korea; 6 The Way Communications, Seoul, Republic of Korea; 7 Division of Cardiology, Department of Internal Medicine, Hallym University Sacred Heart Hospital, 22 Gwanpyeong-ro 170-beon-gil, Dongan-gu, Anyang, 14068 Republic of Korea; 8 Department of Cardiology, Cardiovascular Center, Korea University Anam Hospital, 126-1, 5ka, Anam-dong, Sungbuk-ku, Seoul, 136-705 Republic of Korea

**Keywords:** Cardiology, Hypertension, Prognosis

## Abstract

Although both angiotensin receptor blockers (ARBs) and dihydropyridine calcium channel blockers (CCBs) are all suitable for the initiation of antihypertensive treatment, studies investigating efficacy and safety between ARBs and CCBs are limited, and there is no previous study comparing their clinical outcomes during long-term follow-up periods in real world setting. We compared cardiovascular (CV) events between ARBs and CCBs in 464,948 hypertensive adults using the Korean National Health Insurance Service database during a 3-year follow-up. The patients with hypertension without heart failure, ischemic heart disease, cerebrovascular disease, or peripheral artery disease were enrolled. The CV events between only single prescription of CCBs and ARBs were finally compared. The primary endpoint for this study was the first occurrence of a major adverse CV events, defined as the composite of all-cause death, cardiac death, nonfatal myocardial infarction, or nonfatal stroke. ARB was significantly more administered in male and patients with higher income, diabetes mellitus, chronic kidney diseases, and higher Charlson comorbidity index. The primary endpoints occurred in 10,526 patients (5.2%) in the ARB group and in 19,363 patients (7.3%) in the CCB group (p < 0.001) during a 3-year follow-up (HR 0.96, 95% CI 0.93–0.98). All the components of CV events including all-cause death, cardiac death, nonfatal myocardial infarction, and nonfatal stroke occurred more frequently in the CCB group. With multivariable models adjusting age, sex, income, diabetes, chronic kidney disease, and Charlson comorbidity index, the primary endpoints less frequently developed in the ARB group than in the CCB group (HR 0.957, 95% CI 0.933–0.983, p < 0.001). After the propensity-score matching, baseline characteristics were similar and still showed significantly better primary endpoints in ARB group than CCB group (5.3% vs. 5.8%, p < 0.001). In this nationwide population-based simple hypertension study, administration of ARBs showed superior protection against CV events than CCBs during a 3-year follow-up. Our results suggest that ARBs could be preferred over CCBs as the initial choice of antihypertensive treatment regardless of age in real-world practice.

## Introduction

The number of hypertensive patients has been increasing and is expected to increase further worldwide. The objectives of antihypertensive treatment are to prevent future cardiovascular (CV) adverse events from sustained high blood pressure (BP)^1,2^. Antihypertensive drugs have demonstrated to improve clinical outcomes and prolong life by myriads of clinical trials. Among those previous trials, renin–angiotensin-system (RAS) inhibitors and calcium channel blockers (CCBs) are the most widely investigated antihypertensive drugs and have concrete data on improving mortality and morbidity by effectively lowering blood pressure. Although improved clinical outcomes by antihypertensive drugs were attributed mainly to BP reduction itself, pleiotropic effects of different antihypertensive drugs such as improvement in endothelial function and anti-inflammatory effects have been suggested for influencing clinical events. Angiotensin receptor blockers (ARBs) are the most frequently prescribed antihypertensive drug followed by CCBs in Korea. However, studies investigating efficacy and safety between ARBs and CCBs are limited, and there is no previous study comparing their clinical outcomes during long-term follow-up periods in real world setting. Therefore, we investigated the clinical impacts of ARBs and CCBs on CV events in 464,948 simple hypertensive adults using the Korean National Health Insurance Service (NHIS) database during a 3-year follow-up.

## Methods

### Data sources and study patients

We compared the clinical impact of ARBs and CCBs on CV events by using the Korean NHIS cohort data. The NHIS is a mandatory national health coverage system which all citizens in South Korea have to join. It had 1.3 trillion cases of treatment details and clinic status^3^. The Korea University Anam Hospital Institutional Review Board approved this study, and the requirement for informed consent was waived because the NHIS database was constructed after anonymization according to strict confidentiality guidelines. All methods were performed in accordance with relevant guidelines and regulations.

**Figure 1:**
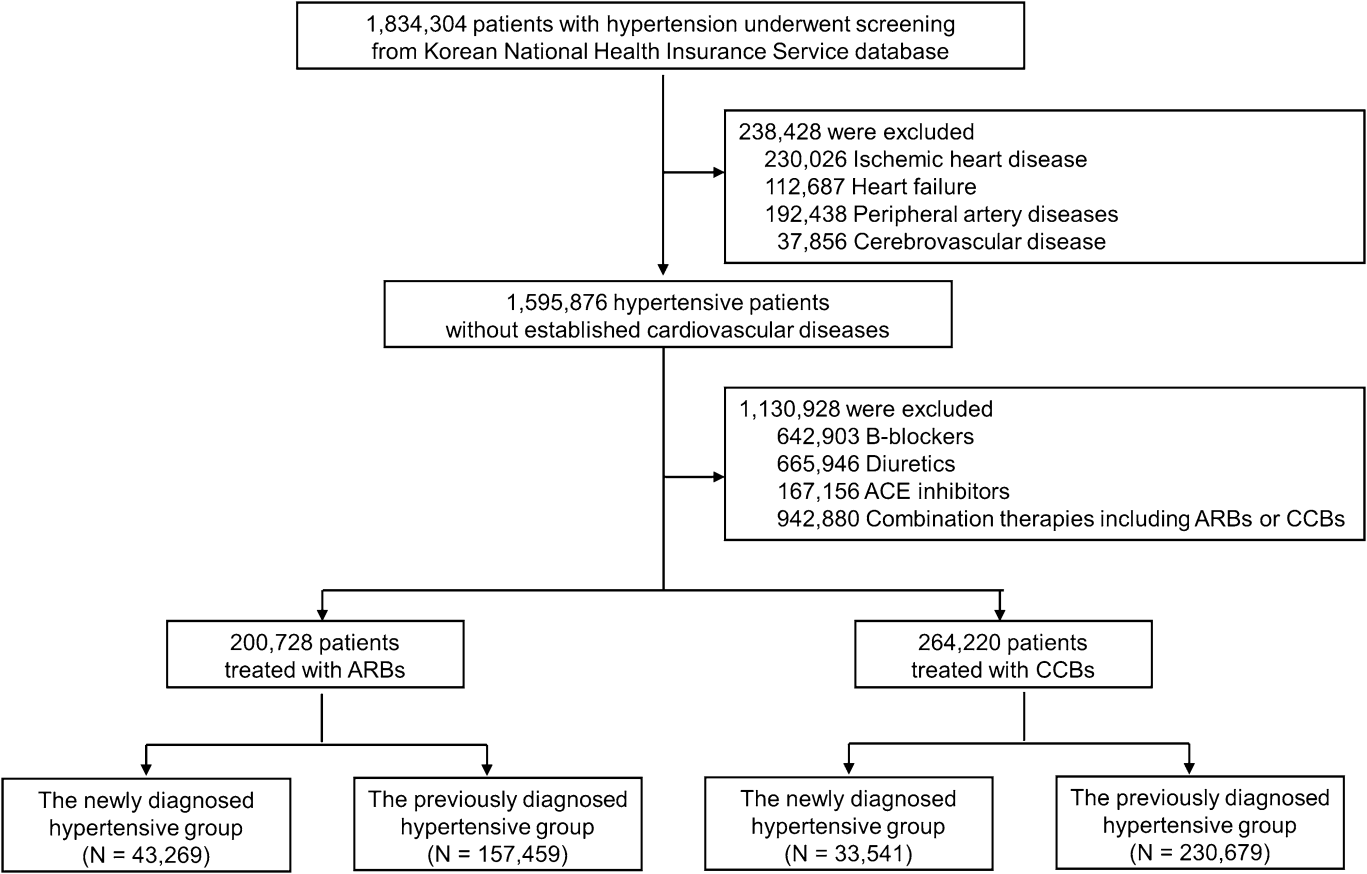
Study protocol.

Among 1,834,304 patients ≥ 20 years from the 2013 NHIS cohort, patients with hypertension were evaluated (Fig. 1). Hypertension was defined as I10 of International Classification of Diseases-10th Revision (ICD-10) codes. Patients were excluded who had a history of heart failure (HF) (I50, I110, I130, I132 codes), ischemic heart disease (I20-I25 codes), cerebrovascular disease (I61-I69 codes), or peripheral artery disease (I732, I738, I739, I771, I790, I792, K551, K558, K559, K551, K558, K559, Z958, Z959 codes). Those whose prescription records were not identified were also excluded. The patients administered with b-blockers, diuretics, and ACE inhibitors were excluded. In addition, combination therapies including ARBs or CCBs were also excluded. Only the patients on monotherapy with ARBs or CCBs were included in this study. The incidence of CV events in the screened patients was finally compared between single use of CCB and ARB. The frequency of the drugs according to the types and dosage were described at Supplementary Table S1.

Both groups were divided into the newly diagnosed hypertensive group and the previously diagnosed hypertensive group. The newly diagnosed hypertensive group was defined if the patients had no ICD-10 codes related to hypertension and did not received any anti-hypertensive drugs at the screening. The first treatment drug was defined as the longest prescribed anti-hypertensive drug during the observed period. If the longest treatment drug was prescribed shorter than 7 days after 90 days of the first diagnosis, those cases were excluded from this study. The previously diagnosed hypertensive group was defined if the patients were already diagnosed with hypertension at the screening. The primary therapy drug was defined as the longest anti-hypertensive drug more than 30% of 3-year follow-up. The patients whose administration period less than 329 days were excluded from this study.

### Endpoints

The primary endpoint for this study was the first occurrence of a major adverse cardiovascular events (MACEs), defined as the composite of all-cause death, cardiac death, nonfatal myocardial infarction (MI), or nonfatal stroke. Secondary endpoints were each component of all-cause death, MI, revascularization, admission from HF, and ischemic stroke. Events were assessed until December 31, 2016 after 3-year follow up from 2013.

In our study, hypertension was defined according to ICD-10 codes by physician`s diagnosis. All-cause death was identified by the presence of death date in data. Cardiac death includes sudden cardiac death, death due to acute MI, death due to stroke, death due to heart failure, and death due to other CV causes (ICD-10 codes of I21, I61, I62, I63, I64, I50, I130, I132, I110, I46). Other events were ascertained according to the principal diagnosis of hospital admissions on the basis of ICD-10 codes (for MI, I21, I22; stroke, I61–I69; ischemic stroke, I63, I64). Revascularization was defined when the medical records of percutaneous coronary intervention or coronary artery bypass graft surgery were identified during follow-up periods. Admission from heart failure was defined when the patients with heart failure symptoms were admitted and treated with diuretics.

### Statistical analysis

The patients` characteristics and comorbidities were summarized in each group. Data were expressed as mean ± standard deviation for the continuous variables, and as number and percentage of patients for the categorical variables. Fisher’s exact test or Chi-square test was used for categorical variables. A Cox proportional hazards model was used to test for trends in the incidence of adverse CV events. To adjust potential confounding factors for MACEs, multivariable models were applied for stratified age at baseline, sex, income, diabetes, chronic kidney disease, and Charlson comorbidity index (CCI). Stratified analyses of MACEs for subgroups of age, gender, income, diabetes mellitus, chronic kidney disease, and Charlson comorbidity index were conducted and compared using p values for interaction. To balance the distribution of baseline characteristics, we used propensity score-matching. We estimated a propensity score for each study participant using the multivariable logistic regression model. In the model, potential confounders and variables, such as age, gender, income, diabetes mellitus, chronic kidney disease, and Charlson comorbidity index were included. We then created an exchangeable comparison group of patients with ARB group by matching each with CCB group in a 1:1 fashion with a caliper of 0.008. The model was fit to the data during all steps of the regression analyses (Wald test, p < 0.001). Using the propensity score, we matched 162,446 patients with ARBs to another 162,446 patients with CCBs who had a similar propensity score. After matching, the mean propensity score for the patients using ARBs and CCBs were 0.44 ± 0.12 and 0.44 ± 0.12, respectively. Furthermore, we have conducted a sensitivity analysis with E-value methods for assessing robust associations among potential unmeasured confounders. Higher E-value means considerable unmeasured confounders would be present^4^. p value < 0.05 was considered statistically significant. SAS software (version 9.3; SAS Institute, Cary, NC, USA) was used for statistical analysis.

### Ethics approval and consent to participate

The Korea University Anam Hospital Institutional Review Board approved this study, and the requirement for informed consent was waived because the NHIS database was constructed after anonymization according to strict confidentiality guidelines.

## Results

### Patient characteristics

Baseline patient characteristics between the ARB and CCB groups were significantly different (Table 1). ARBs were more preferred in younger patients than CCBs. In addition, ARBs were administrated more frequently in male and patients with higher income, DM, CKD, and higher CCI. These prescribing patterns of CCBs and ARBs were consistent within the newly diagnosed hypertensive group and the previously diagnosed hypertensive group (Supplementary Tables S2, S3). Mean usage time of ARB was 1220.3 ± 524.3 days and CCBs 1313.7 ± 553.5 days (p < 0.001). After propensity score matching, baseline characteristics were similar between two groups (Supplementary Table S4). Baseline and follow-up mean BP after the anti-hypertensive therapy in the first treatment group were significantly higher in ARB group than CCB group (Supplementary Table S5). However, systolic BP at baseline and follow-up in the primary therapy group was similar between two groups. Target BP achievement rate (SBP/DBP ≤ 140/90) were significantly higher in CCB group.

**Table 1:** Baseline demographic characteristics.

Variable	ARB (n = 200,728)	CCB (n = 264,220)	p-value
**Age (years)**			< 0.001
< 40	8230 (4.1)	4667 (1.8)	
40–49	33,374 (16.6)	22,136 (8.4)	
50–59	68,605 (34.2)	67,529 (25.6)	
60–69	50,448 (25.1)	75,574 (28.6)	
70-	40,071 (20.0)	94,314 (35.7)	
Female	102,980 (51.3)	155,582 (58.9)	< 0.001
**Income**			< 0.001
< 25%	29,317 (15.4)	41,289 (16.6)	
25–75%	76,426 (40.1)	98,761 (39.7)	
> 75%	84,727 (44.5)	108,933 (43.8)	
Diabetes mellitus	34,451 (17.2)	23,845 (9.4)	< 0.001
Chronic kidney disease	1183 (0.6)	431 (0.2)	< 0.001
**Commodity channel index**			< 0.001
0	90,398 (46.6)	124,221 (47.9)	
1	65,058 (33.5)	84,569 (32.6)	
2	26,278 (13.5)	34,193 (13.2)	
> 3	12,345 (6.4)	16,508 (6.4)	

Values are presented as mean ± standard deviation or n (%).

*ARB* angiotensin receptor blocker, *CCB* calcium channel blocker.

### Clinical outcomes at 3-year follow-up

**Figure 2:**
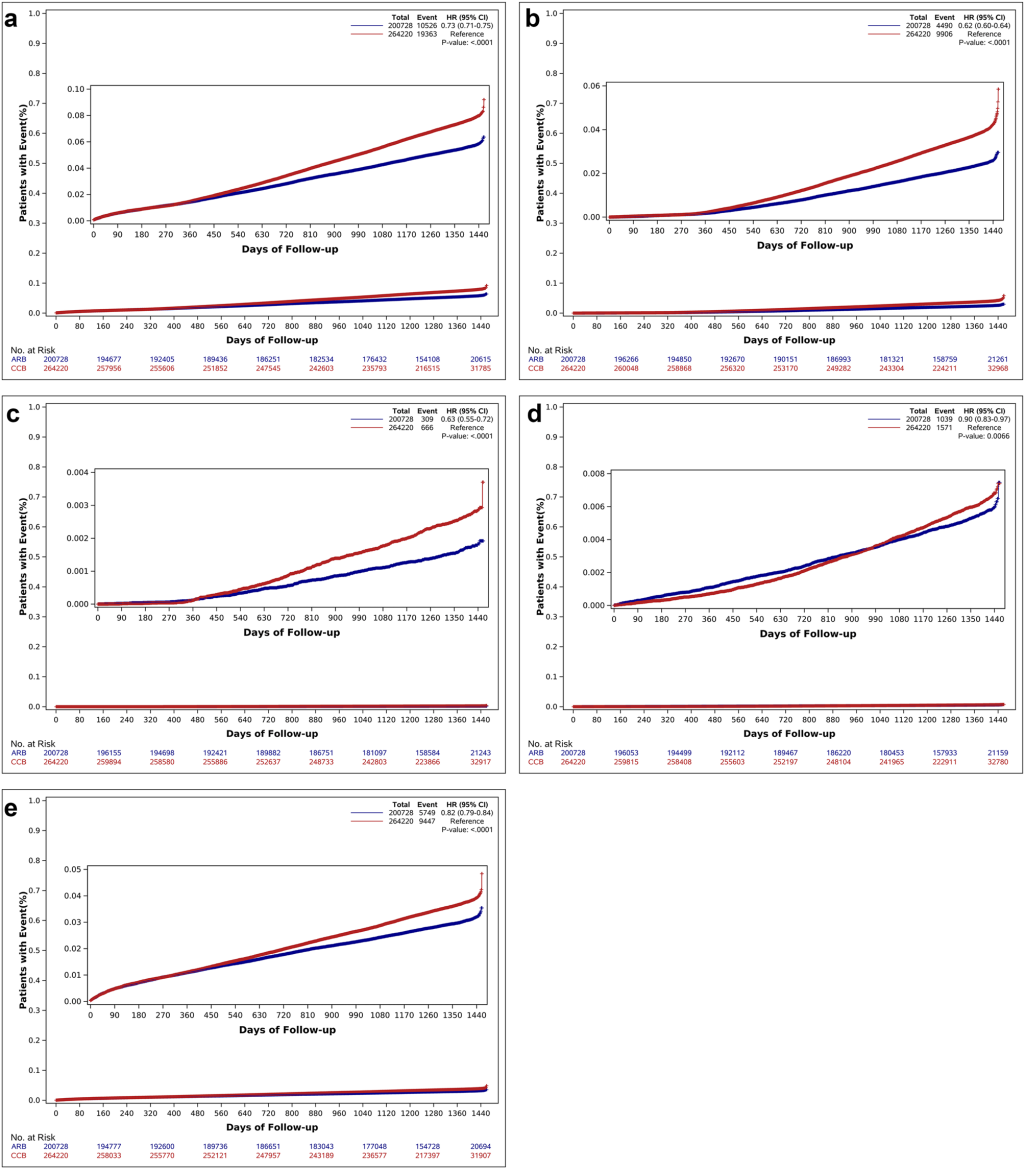
Cumulative incidence rates of major adverse cardiovascular events during a 3-year follow-up. (**a**) Major adverse cardiovascular events. (**b**) All cause death. (**c**) Cardiac death. (**d**) nonfatal MI. (**e**) Stroke.

The primary endpoints occurred in 5.2% (10,526/200,728 patients) in the ARB group and in 7.3% (19,363/264,220 patients) in the CCB group (HR 0.73, 95% CI 0.71–0.75, p < 0.001) during 3-year follow-up (Table 2, Fig. 2a–e). All the components of MACEs including all-cause death (HR 0.62, 95% CI 0.60–0.64, p < 0.001), cardiac death (HR 0.63, 95% CI 0.55–0.72, p < 0.001), nonfatal MI (HR 0.90, 95% CI 0.83–0.97, p = 0.007), and stroke (HR 0.82, 95% CI 0.79–0.84, p < 0.001) occurred more frequently in the CCB group. In addition, the incidence of the secondary composite endpoint including all-cause death, nonfatal MI, admission from HF, revascularization, and ischemic stroke was significantly higher in CCB group (8.8% vs. 11.0%, p < 0.001). In analyses of the separate clinical events, incidences of each event in CCB group were significantly higher than in ARB group. However, there were similar rates of revascularization in the ARB group and CCB group. The HR of ARBs over the CCBs for the primary MACEs was 0.957 (95% CI 0.933–0.983). The RR value using HR is 0.970 (95% CI 0.953–0.988), and the E-value is 1.210 (95% CI 1.122–1.277). After propensity score matching, the primary and secondary MACEs were significantly lower in ARB group (Supplementary Table S6, Supplementary Fig. S1).

**Table 2:** Incidence of clinical events during a 3-year follow-up.

Variable	ARB (n = 200,728)	CCB (n = 264,220)	p-value
**Primary outcome (MACEs)**	10,526 (5.2)	19,363 (7.3)	< 0.001
All cause death	4490 (2.2)	9906 (3.7)	< 0.001
Cardiac death	309 (0.2)	666 (0.3)	< 0.001
Nonfatal myocardial infarction	1039 (0.5)	1571 (0.6)	< 0.001
Nonfatal stroke	5749 (2.9)	9447 (3.6)	< 0.001
**Secondary outcome**	17,634 (8.8)	29,139 (11.0)	< 0.001
All cause death	4490 (2.2)	9906 (3.7)	< 0.001
Nonfatal myocardial infarction	1039 (0.5)	1571 (0.6)	< 0.001
Revascularization	1621 (0.8)	2027 (0.8)	0.122
Admission from heart failure	947 (0.5)	1728 (0.7)	< 0.001
Ischemic stroke	5035 (2.5)	8283 (3.1)	< 0.001

Values are presented as n (%).

*ARB* angiotensin receptor blocker, *CCB* calcium channel blocker.

**Figure 3:**
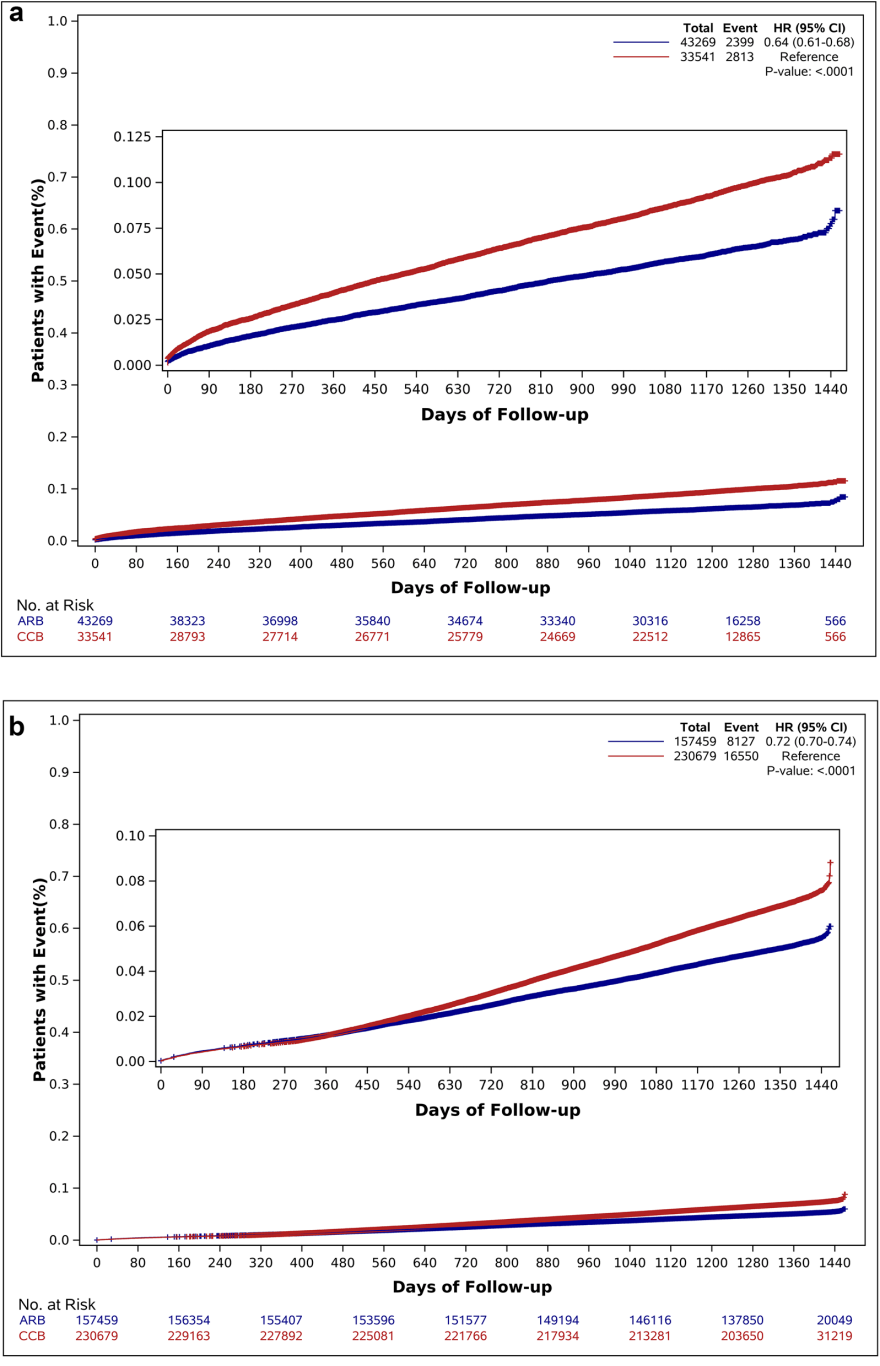
Hazard ratios and 95% confidence intervals of major adverse cardiovascular events. (**a**) The newly diagnosed hypertensive group. (**b**) The previously diagnosed hypertensive group.

**Figure 4:**
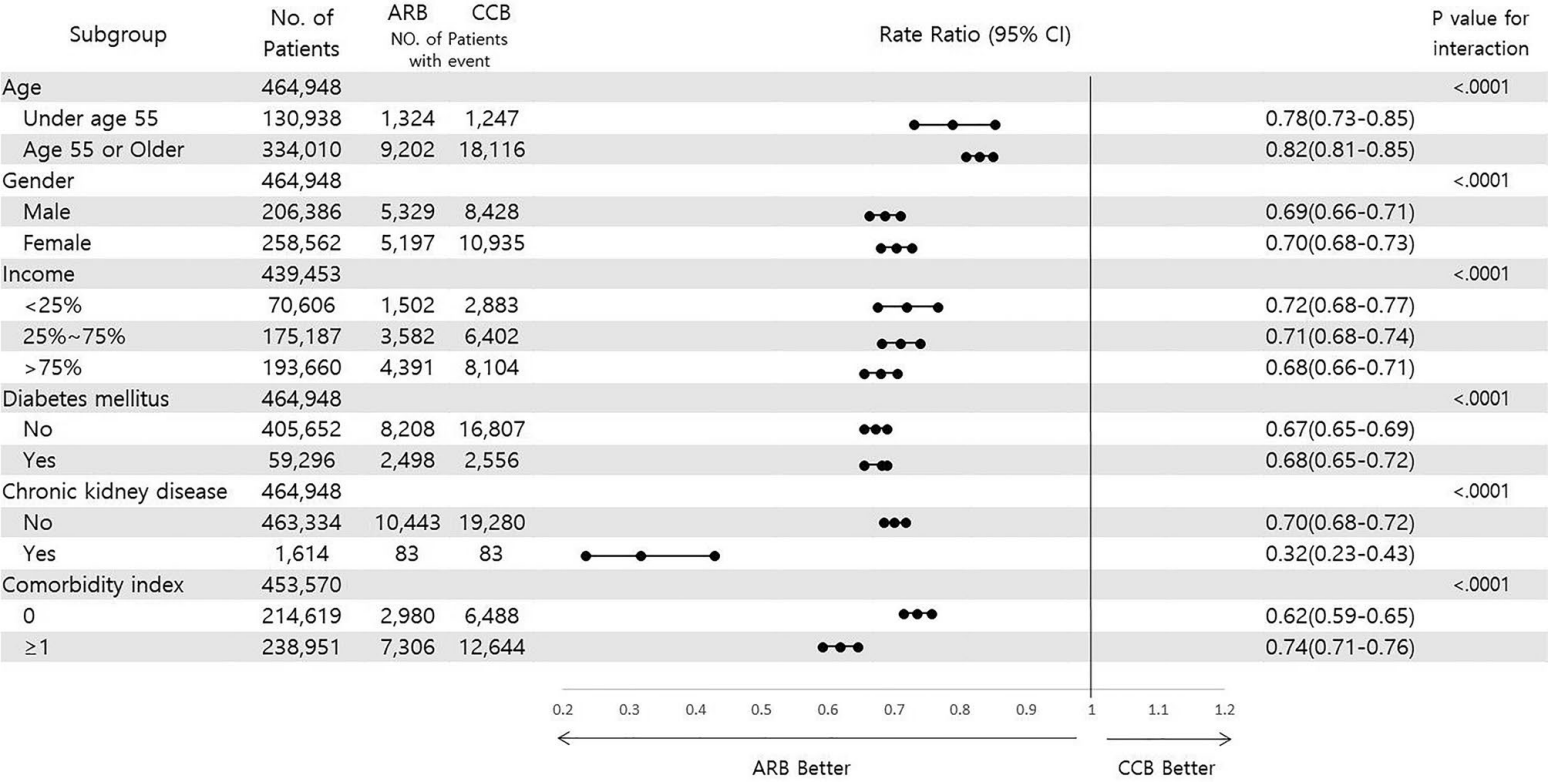
Prespecified subgroup analyses of MACEs.

Both the newly diagnosed hypertensive group (HR 0.64, 95% CI 0.61–0.68, p < 0.001) and the previously diagnosed hypertensive group (HR 0.72, 95% CI 0.70–0.74, p < 0.001) showed significantly higher incidences of primary outcomes by the administration of CCBs (Supplementary Tables S7, S8, Fig. 3a,b). The secondary outcomes were also more frequent in both the newly diagnosed hypertensive group and the previously diagnosed hypertensive group with CCBs than ARBs. Noticeably, these adverse events more frequently occurred in the CCB group regardless of age criteria of under and more than 55 years (Supplementary Tables S9, S10). In subgroup analyses of MACEs, better clinical outcomes were consistently observed in ARB group across all prespecified subgroups (Fig. 4).

### Predictors for MACEs

With multivariable models adjusting age, sex, income, diabetes, chronic kidney disease, and CCI, MACEs developed less frequently in the ARB group than in the CCB group (HR 0.96, 95% CI 0.93–0.98, p < 0.001) (Table 3). Irrespective of the newly diagnosed hypertensive group or the previously diagnosed hypertensive group, administration of ARBs significantly decreased the risks of MACEs (Supplementary Tables S11, S12). In comparison with CCBs, ARBs lowered the risks of MACEs by 30% and 19% in patients under age 55 and over 55, respectively (Supplementary Tables S13, S14).

**Table 3:** Predictors for MACEs.

Risk Factor	Univariate analysis				Multivariate analysis			
	HR	95% CI		p-value	HR	95% CI		p-value
		Lower	Upper			Lower	Upper	
ARB	0.732	0.715	0.750	< 0.001	0.957	0.933	0.983	0.001
**Age**								
< 40	1				1			
40–49	1.408	1.179	1.681	< 0.001	1.452	1.190	1.770	< 0.001
50–59	2.044	1.725	2.241	< 0.001	2.174	1.799	2.627	< 0.001
60–69	3.609	3.051	4.270	< 0.001	3.785	3.135	4.569	< 0.001
70–	11.161	9.446	13.188	< 0.001	11.894	9.862	14.344	< 0.001
Female	0.906	0.886	0.927	< 0.001	0.701	0.684	0.719	< 0.001
**Income**								
< 25%	1				1			
25–75%	0.922	0.890	0.956	< 0.001	0.988	0.953	1.024	0.499
> 75%	1.039	1.004	1.075	0.029	0.892	0.610	0.924	< 0.001
Diabetes mellitus	1.394	1.353	1.437	< 0.001	1.083	1.048	1.120	< 0.001
Chronic kidney disease	1.776	1.525	2.069	< 0.001	1.332	1.130	1.572	0.001
**Comorbidity channel index**								
0	1				1			
1	1.507	1.465	1.550	< 0.001	1.305	1.266	1.345	< 0.001
2	2.087	2.019	2.157	< 0.001	1.616	1.559	1.674	< 0.001
> 3	3.436	3.314	3.563	< 0.001	2.360	2.268	2.455	< 0.001

*ARB* angiotensin receptor blocker, *CCB* calcium channel blocker.

## Discussion

This nationwide population-based study of about half million adults compared the clinical outcomes between the ARBs and CCBs during a 3-year follow-up in hypertensive patients. Administration of ARBs significantly reduced the incidences of MACEs compared to CCBs. ARBs showed superior protection against CV events than CCBs regardless of the newly diagnosed hypertensive group or the previously diagnosed hypertensive group. This is the first study, to our knowledge, to show that ARBs were associated with decreased CV events among simple hypertensive patients.

### Clinical evidences of ARBs comparing with CCBs

According to current guidelines of hypertension, 4 classes of antihypertensive drugs including RAS inhibitors, beta-blockers, CCBs, and diuretics have been recommended to reduce future CV events^5,6^. The selection of antihypertensive drugs can be dependent on the patient`s ethnicity, age, or preferred indications^7,8^. In black patients, thiazide diuretics or CCBs showed greater BP-lowering effects compared with RAS inhibitors or beta-blockers, contributing to reduction in adverse CV events^9^. In National Institute for Health and Care Excellence (NICE) guideline, CCBs is recommended as the first choice of initial antihypertensive therapy in patients older than 55 years old or in black patients^10^. Moreover, CCBs are preferred in patients with isolated systolic hypertension or ischemic heart disease^1,11^. Based on previous trials, RAS inhibitors are indicated in hypertensive patients with HF, diabetes, chronic kidney disease, or post-MI^1,11^. However, the side effects of ACE inhibitors such as dry cough were more common in Asians than Western people, relating to the high discontinuation rates of ACE inhibitors^12^. Therefore, prescription of ARBs for hypertension is more than 20-folds when compared to ACE inhibitors (24.5% vs. 1.5%) during monotherapy in Korea^13^. Both ARBs and CCBs could be used as the first-line anti-hypertensive medications. However, there was no clear evidence in the simple hypertensive patients without preferred indications, and there are only a few studies comparing ARBs with CCBs according to the age. ARBs, when compared to CCBs, revealed consistent benefit of reducing CV events in patients less than 55 years and older than 55 years old in our study. Although several randomized trials comparing ARBs with CCBs have been conducted, simple hypertensive patients were excluded in those trials, and included patients were heterogeneous. The Valsartan Antihypertensive Long-term Use Evaluation (VALUE) trial including 15,245 patients and the Morbidity and Mortality After Stroke, and the Candesartan Antihypertensive Survival Evaluation in Japan Trial (CASE-J) including 4703 patients showed similar rates of CHD, CV death, and all-cause death^14–17^. However, the primary composite cardiovascular outcome of the Irbesartan Diabetic Nephropathy Trial (IDNT) including 1146 patients and Eprosartan Compared with Nitrendipine for Secondary Prevention (MOSES) study including 1405 patients were significantly lower in ARBs. Nonetheless, a previous meta-analysis including those previously mentioned studies reported significantly higher incidences for stroke and MI in ARBs than CCBs^18^. Nonetheless, there were some argues in that further analysis with a random-effect model showed similar MI risks between two groups. Although MOSES trial targeting the patients with a prior stroke within 24 months showed more cerebrovascular events in CCBs than ARBs, the trial was excluded in the meta-analysis. In addition, NAGOYA HEART study which was included in this meta-analysis has been retracted for the wrong event definition. Compared to previous studies, one of the main strengths of our study is that our study is the first large-scale study comparing ARBs with CCBs during long-term follow-up periods in real-world setting. Our large-scale trial targeted simple hypertensive patients without CV diseases, showing superior CV protection of ARBs against CCBs despite of having relatively higher risk factors in ARB group such as higher rates of DM, CKD, and higher Charlson comorbidity index scores. Similar to our study, the Efficacy of Candesartan on Outcome in Saitama Trial (E-COST) which targeted simple hypertensive patients demonstrated that candesartan compared to conventional treatment of mainly CCBs significantly reduced the incidence of stroke by 39% and MI by 57%^19^.

### Pathophysiological evidences of ARBs comparing with CCBs

In a real-world practice study, ARBs reported 10% lower rates of CV events compared to ACE inhibitors in patients with established CV disease during 4-year follow-up^20^. AT1 receptor blockers by ARBs induce a dose-dependent blockade of angiotensin II-induced effects, resulting in a reduction in BP, cardiac and vascular hypertrophy, proteinuria and glomerular sclerosis^21,22^. It is postulated that ARBs may provide end-organ protection by blocking angiotensin II via the AT1 receptor and enhance vasodilatory effects by mediating increases in renal interstitial fluid bradykinin concentrations^21,22^. In previous studies, it has been suggested that ARBs would have more benefits on cardiovascular functions than CCBs. In patients with left ventricular hypertrophy (LVH), a significantly larger decrease in left ventricular mass index was observed in ARB group than in CCB group^23^. It was reported that the RAS-inhibiting agents revealed the greatest regressive effects of LVH among different antihypertensive agents, and the direct action of RAS inhibition rather than the BP-lowering effect contributed considerably to the regression of LVH^23,24^. The preventive effects of RAS inhibitors on new-onset diabetes compared to CCBs also have been reported by large-scale clinical trials^25^. Regression of LVH and attenuation of new-onset diabetes were associated with a reduction of CV events^26,27^. In addition, ARBs improved coronary flow velocity reserve in hypertensive patients, which was not observed in the CCB group^28^. ARBs were more effective than CCBs for potentially ameliorating atherosclerosis by decreasing brachial-ankle pulse wave velocity and intima-media thickness^29^. With these postulated mechanisms, our results suggest that ARBs could provide superior protection against CV events than CCBs as the initial choice of antihypertensive treatment in simple hypertensive patients in real-world practice. Based on ARBs as the initial therapy, add-on therapy with different antihypertensive classes for other indications or more BP reduction can be a favorable strategy.

### Limitations

To our knowledge, this is the largest real-world study comparing ARBs with CCBs during long-term follow-up periods, showing superior protection of ARBs against CV events than CCBs. However, this study has a few limitations. First, smoking, dyslipidemia, and adherence data to therapy which were taken into account were not available in this study. Second, the duration of hypertension could not be assessed. Third, since the study population was from a single country, the results may not necessarily be generalizable to people of other racial or ethnic backgrounds. Forth, we did not evaluate other medications outside of antihypertensive agents; however, since we included only simple hypertensive patents in this study, the effects of non-hypertensive medications would be very limited in such a large scale study. Finally, there was also no data available on adherence to reduced salt intake or other dietary recommendations, as well as type of physical activity, which may be important confounders in the association of blood pressure with CVD. With about half million patients included in this nationwide cohort study, lifestyle modifications including dietary and physical measures would equilibrate between the two groups.

## Conclusions

In this nationwide population-based study of 464,948 simple hypertensive adults, administration of ARBs showed superior protection against CV events than CCBs during a 3-year follow-up. Our results suggest that ARBs could be used as the initial choice of antihypertensive treatment regardless of age in real-world practice. Based on ARBs as the initial therapy, add-on therapy with other antihypertensive classes for other indications or more BP reduction can be a favorable strategy in simple hypertensive patients.

## Supplementary Information

Supplementary Information 1.

Supplementary Information 2.

Supplementary Information 3.

Supplementary Information 4.

Supplementary Information 5.

Supplementary Information 6.

Supplementary Information 7.

## Data Availability

Because we have analyzed the Korean National Health Insurance Service database, data sharing policy dependents on the Korean National Health Insurance Service. The health information data are only provided by Statistic analysis tool in “Data analysis room” located within the National Health Insurance Corporation in which PC for review and analysis of data is installed.

## Abbreviations

BP: Blood pressure
CCB: Calcium channel blockers
CCI: Charlson comorbidity index
CV: Cardiovascular
ICD-10: International Classification of Diseases-10th Revision
HF: Heart failure
LVH: Left ventricular hypertrophy
MACE: Major adverse cardiovascular event
MI: Myocardial infarction
NHIS: National Health Insurance Service
RAS: Renin-angiotensin-system
