# Supplementary material for: Clinical outcomes between calcium channel blockers and angiotensin receptor blockers in hypertensive patients without established cardiovascular diseases during a 3-year follow-up
Source: Sci Rep. 2021 Jan 19;11:1783. doi: 10.1038/s41598-021-81373-7 (PMC7815918; doi:10.1038/s41598-021-81373-7)
Supplement: Supplementary file 1 — Supplementary Information 1. [file 41598_2021_81373_MOESM1_ESM.docx]

**Clinical Outcomes between Calcium Channel Blockers and Angiotensin Receptor Blockers in Hypertensive Patients without Established Cardiovascular Diseases during a 3-Year Follow-up**

Han Saem Jeong, MD^1, *^; Hong‐Seok Lim, MD^2, *^; Hun-Jun Park, MD^3^; Wang-Soo Lee, MD^4^; Jin-Oh Choi, MD^5^; Hui Seung Lee^6^; Sang-Ho Jo, MD^7, §^; Soon Jun Hong, MD^8, §^

**Supplementary Figure 1.** Cumulative incidence rates of major adverse cardiovascular events during a 3-year follow-up after propensity score matching

(A) Major adverse cardiovascular events

(B) All cause death

(C) Cardiac death

(D) nonfatal MI

(E) Stroke
