# Supplementary material for: Clinical outcomes between calcium channel blockers and angiotensin receptor blockers in hypertensive patients without established cardiovascular diseases during a 3-year follow-up
Source: Sci Rep. 2021 Jan 19;11:1783. doi: 10.1038/s41598-021-81373-7 (PMC7815918; doi:10.1038/s41598-021-81373-7)
Supplement: Supplementary file 2 — Supplementary Information 2. [file 41598_2021_81373_MOESM2_ESM.docx]

**Clinical Outcomes between Calcium Channel Blockers and Angiotensin Receptor Blockers in Hypertensive Patients without Established Cardiovascular Diseases during a 3-Year Follow-up**

Han Saem Jeong, MD^1, *^; Hong‐Seok Lim, MD^2, *^; Hun-Jun Park, MD^3^; Wang-Soo Lee, MD^4^; Jin-Oh Choi, MD^5^; Hui Seung Lee^6^; Sang-Ho Jo, MD^7, §^; Soon Jun Hong, MD^8, §^

**Supplementary Table 1.** Frequency of the drugs according to the types and dosage

| Drug | ARB | | |
| --- | --- | --- | --- |
|  | (n=200,728) | | |
|  | n | % | |
| candesartan cilexetil 8mg | 13,209 | 6.58 | |
| candesartan cilexetil 16mg | 6,863 | 3.42 | |
| candesartan cilexetil 32mg | 198 | 0.10 | |
| eprosartan mesylate (as eprosartan 600mg) | 1,440 | 0.72 | |
| fimasartan potassium 30mg | 2,593 | 1.29 | |
| fimasartan potassium 60mg | 10,866 | 5.41 | |
| fimasartan potassium 120mg | 1,744 | 0.87 | |
| irbesartan 150mg | 10,398 | 5.18 | |
| irbesartan 300mg | 963 | 0.48 | |
| losartan potassium (as losartan 45.8mg) | 58,619 | 29.20 | |
| losartan potassium (as losartan 91.6mg) | 5,705 | 2.84 | |
| olmesartan medoxomil 10mg | 5,053 | 2.52 | |
| olmesartan medoxomil 20mg | 19,912 | 9.92 | |
| olmesartan medoxomil 40mg | 446 | 0.22 | |
| telmisartan 40mg | 26,666 | 13.28 | |
| telmisartan 80mg | 6,501 | 3.24 | |
| valsartan 40mg | 1,833 | 0.91 | |
| valsartan 80mg | 22,199 | 11.06 | |
| valsartan 160mg | 5,499 | 2.74 | |
| valsartan 320mg | 21 | 0.01 | |
| Drug | CCB | |  |
|  | (n=264,220) | |  |
|  | n | % |  |
| amlodipine adipate (as amlodipine 5mg) | 5,218 | 1.97 |  |
| amlodipine adipate (as amlodipine 10mg) | 27 | 0.01 |  |
| amlodipine besylate (as amlodipine 5mg) | 88,237 | 33.40 |  |
| amlodipine besylate (as amlodipine 6.944mg) | 80 | 0.03 |  |
| amlodipine besylate (as amlodipine 10mg) | 2,704 | 1.02 |  |
| amlodipine besylate (as amlodipine 13.888mg) | 5 | 0.00 |  |
| amlodipine camsylate (as amlodipine 5mg) | 25,438 | 9.63 |  |
| amlodipine camsylate (as amlodipine 10mg) | 50 | 0.02 |  |
| amlodipine maleate (as amlodipine 2.5mg) | 186 | 0.07 |  |
| amlodipine maleate (as amlodipine 5mg) | 40,754 | 15.42 |  |
| amlodipine maleate (as amlodipine 10mg) | 4 | 0.00 |  |
| amlodipine mesylate monohydrate (as amlodipine 5mg) | 2,154 | 0.82 |  |
| amlodipine mesylate monohydrate (as amlodipine 10mg) | 99 | 0.04 |  |
| amlodipine nicotinate (as amlodipine 5mg) | 2,664 | 1.01 |  |
| amlodipine orotate (as amlodipine 5mg) | 11,662 | 4.41 |  |
| amlodipine oratate (as amlodipine 10mg) | 7 | 0.00 |  |
| barnidipine hydrochloride 5mg | 210 | 0.08 |  |
| benidipine hydrochloride 2mg | 210 | 0.08 |  |
| benidipine hydrochloride 4mg | 2,378 | 0.90 |  |
| benidipine hydrochloride 6mg | 183 | 0.07 |  |
| benidipine hydrochloride 8mg | 685 | 0.26 |  |
| cilnidipine 5mg | 1,248 | 0.47 |  |
| cilnidipine 10mg | 3,734 | 1.41 |  |
| diltiazem hydrochloride 30mg | 508 | 0.19 |  |
| diltiazem hydrochloride 90mg | 2,214 | 0.84 |  |
| diltiazem hydrochloride 180mg | 679 | 0.26 |  |
| efonidipine 20mg | 102 | 0.04 |  |
| efonidipine 40mg | 366 | 0.14 |  |
| felodipine 2.5mg | 484 | 0.18 |  |
| felodipine 5mg | 11,557 | 4.37 |  |
| isradipine 5mg | 657 | 0.25 |  |
| lacidipine 2mg | 1,175 | 0.44 |  |
| lacidipine 4mg | 2,288 | 0.87 |  |
| lacidipine 6mg | 221 | 0.08 |  |
| lercanidipine hydrochloride 10mg | 12,055 | 4.56 |  |
| lercanidipine hydrochloride 20mg | 780 | 0.30 |  |
| manidipine hydrochloride 10mg | 791 | 0.30 |  |
| manidipine hydrochloride 20mg | 457 | 0.17 |  |
| nicardipine hydrochloride 20mg | 21 | 0.01 |  |
| nicardipine hydrochloride 40mg | 42 | 0.02 |  |
| nifedipine 5mg | 72 | 0.03 |  |
| nifedipine 10mg | 6 | 0.00 |  |
| nifedipine 33mg | 5,658 | 2.14 |  |
| nifedipine 40mg | 320 | 0.12 |  |
| nifedipine(micronized) 33mg | 22 | 0.01 |  |
| nifedipine(micronized) 66mg | 960 | 0.36 |  |
| nimodipine 30mg | 165 | 0.06 |  |
| nisoldipine 10mg | 323 | 0.12 |  |
| nisoldipine 20mg | 169 | 0.06 |  |
| S-amlodipine besylate (as S-amlodipine 2.5mg) | 20,864 | 7.90 |  |
| S-amlodipine besylate (as S-amlodipine 5mg) | 2,576 | 0.97 |  |
| S-amlodipine gentisate (as S-amlodipine 3.44mg) | 95 | 0.04 |  |
| S-amlodipine nicotinate (as S-amlodipine 2.5mg) | 7,873 | 2.98 |  |
| S-amlodipine nicotinate (as S-amlodipine 5mg) | 1,402 | 0.53 |  |
| verapamil hydrochloride 180mg | 274 | 0.10 |  |
| verapamil hydrochloride 240mg | 115 | 0.04 |  |
| verapamil hydrochloride 40mg | 149 | 0.06 |  |
| verapamil hydrochloride 80mg | 56 | 0.02 |  |

**Supplementary Table 2.** Baseline demographic characteristics in the newly diagnosed hypertensive group

| Variable | ARB  (n=43,269) | CCB  (n=33,541) | p-value |
| --- | --- | --- | --- |
| Age (years) |  |  | <0.001 |
| <40 | 3,887(9.0) | 2,352(7.0) |  |
| 40-49 | 10,373(24.0) | 5,596(16.7) |  |
| 50-59 | 14,394(33.3) | 9,538(28.4) |  |
| 60-69 | 8,372(19.3) | 7,623(22.7) |  |
| 70- | 6,243(14.4) | 8,432(25.1) |  |
| Female | 19,079(44.1) | 16,288(48.6) | <0.001 |
| Income |  |  | <0.001 |
| < 25% | 6,402(15.6) | 5,217(16.6) |  |
| 25%-75% | 17,846(43.5) | 13,653(43.5) |  |
| > 75% | 16,753(40.9) | 12,524(39.9) |  |
| Diabetes mellitus | 8,165(18.9) | 2,509(7.5) | <0.001 |
| Chronic kidney disease | 318(0.7) | 105(0.3) | <0.001 |
| Commodity channel index |  |  | <0.001 |
| 0 | 21,050(56.7) | 17,452(59.7) |  |
| 1 | 10,702(28.8) | 7,734(26.5) |  |
| 2 | 3,761(10.1) | 2,710(9.3) |  |
| >3 | 1,619(4.4) | 1,336(4.6) |  |

Values are presented as mean ± standard deviation or n (%).

ARB = angiotensin receptor blocker

CCB = calcium channel blocker

**Supplementary Table 3.** Baseline demographic characteristics in the previously diagnosed hypertensive group

| Variable | ARB  (n= 157,459) | CCB  (n= 230,679) | p-value |
| --- | --- | --- | --- |
| Age (years) |  |  | <0.001 |
| <40 | 4,343(2.8) | 2,315(1.0) |  |
| 40-49 | 23,001(14.6) | 16,540(7.2) |  |
| 50-59 | 54,211(34.4) | 57,991(25.1) |  |
| 60-69 | 42,076(26.7) | 67,951(29.5) |  |
| 70- | 33,828(21.5) | 85,882(37.2) |  |
| Female | 83,901(53.3) | 139,294(60.4) | <0.001 |
| Income |  |  | <0.001 |
| < 25% | 22,915(15.3) | 36,072(16.6) |  |
| 25%-75% | 58,580(39.2) | 85,108(39.1) |  |
| > 75% | 67,974(45.5) | 96,409(44.3) |  |
| Diabetes mellitus | 26,286(16.7) | 22,336(9.7) | <0.001 |
| Chronic kidney disease | 865(0.5) | 326(0.1) | <0.001 |
| Commodity channel index |  |  | <0.001 |
| 0 | 69,348(44.2) | 106,769(46.4) |  |
| 1 | 54,356(34.6) | 76,835(33.4) |  |
| 2 | 22,517(14.3) | 31,483(13.7) |  |
| >3 | 10,726(6.8) | 15,172(6.6) |  |

Values are presented as mean ± standard deviation or n (%).

ARB = angiotensin receptor blocker

CCB = calcium channel blocker

**Supplementary Table 4.** Baseline demographic characteristics after propensity score matching

| Variable | ARB  (n=162,446) | CCB  (n=162,446) | p-value |
| --- | --- | --- | --- |
| Age (years) |  |  | 0.99 |
| <40 | 3,898(2.4) | 3,898(2.4) |  |
| 40-49 | 20,032(12.3) | 20,032(12.3) |  |
| 50-59 | 57,619(35.5) | 57,619(35.5) |  |
| 60-69 | 44,971(27.7) | 44,971(27.7) |  |
| 70- | 35,926(22.1) | 35,926(22.1) |  |
| Female | 74,560(45.9) | 74,560(45.9) | 0.99 |
| Income |  |  | 0.99 |
| < 25% | 25,600(15.8) | 25,629(15.8) |  |
| 25%-75% | 65,601(40.4) | 65,617(40.4) |  |
| > 75% | 71,245(43.9) | 71,200(43.8) |  |
| Diabetes mellitus | 20,659(12.7) | 20,659(12.7) | 0.99 |
| Chronic kidney disease | 374(0.2) | 374(0.2) | 0.99 |
| Commodity channel index |  |  | 0.99 |
| 0 | 78,218(48.2) | 78,179(48.1) |  |
| 1 | 53,104(32.7) | 53,069(32.7) |  |
| 2 | 21,129(13.0) | 21,168(13.0) |  |
| >3 | 9,995(6.2) | 10,030(6.2) |  |

Values are presented as mean ± standard deviation or n (%).

ARB = angiotensin receptor blocker

CCB = calcium channel blocker

**Supplementary Table 5.** Mean blood pressure values at baseline and 3-year follow-up.

| Variable | The first treatment group | | p-value | The primary therapy group | | p-value |
| --- | --- | --- | --- | --- | --- | --- |
|  | ARB | CCB |  | ARB | CCB |  |
| Systolic BP at baseline, mmHg | 130.8 ± 14.5 | 129.3 ± 14.3 | <0.001 | 129.5 ± 13.8 | 129.5 ± 13.6 | 0.70 |
| Diastolic BP at baseline, mmHg | 81.2 ± 10.2 | 79.5 ± 9.8 | <0.001 | 79.8 ± 9.5 | 78.8 ± 9.1 | <0.001 |
| Systolic BP at follow-up, mmHg | 130.8 ± 14.3 | 129.3 ± 14.3 | <0.001 | 129.8 ± 13.8 | 129.8 ± 13.6 | 0.97 |
| Diastolic BP at follow-up, mmHg | 81.0 ± 10.0 | 79.3 ± 9.9 | <0.001 | 79.8 ± 9.4 | 78.8 ± 9.1 | <0.001 |
| Target BP achievement rate (%) | 77.3 | 80.2 | <0.001 | 79.6 | 80.7 | <0.001 |

Values are presented as n (%).

ARB = angiotensin receptor blocker

BP = blood pressure

CCB = calcium channel blocker

**Supplementary Table 6.** Incidence of clinical events during a 3-year follow-up after propensity score matching

| Variable | ARB  (n=162,446) | CCB  (n=162,446)) | p-value |
| --- | --- | --- | --- |
| Primary outcome (MACEs) | 8,650(5.3) | 9,437(5.8) | <0.001 |
| All cause death | 3,680(2.3) | 4,484(2.8) | <0.001 |
| Cardiac death | 253(0.2) | 315(0.2) | 0.01 |
| Nonfatal myocardial infarction | 835(0.5) | 921(0.6) | 0.04 |
| Nonfatal stroke | 4,750(2.9) | 4,754(2.9) | 0.97 |
|  |  |  |  |
| Secondary outcome | 14,441(8.9) | 14,481(9.1) | 0.01 |
| All cause death | 3,680(2.3) | 4,484(2.8) | <0.001 |
| Nonfatal myocardial infarction | 835(0.5) | 921(0.6) | 0.04 |
| Revascularization | 1,327(0.8) | 1,191(0.7) | 0.01 |
| Admission from heart failure | 7,231(4.5) | 7,224(4.5) | 0.96 |
| Ischemic stroke | 4,180(2.6) | 4,091(2.5) | 0.32 |

Values are presented as n (%).

ARB = angiotensin receptor blocker

CCB = calcium channel blocker

**Supplementary Table 7.** Incidence of clinical events in the newly diagnosed hypertensive group during a 3-year follow-up

| Variable | The first treatment group | | p-value |
| --- | --- | --- | --- |
|  | ARB  (n=43,269) | CCB  (n=33,541) |  |
| Primary outcome (MACEs) | 2,399(5.5) | 2,813(8.4) | <0.001 |
| All cause death | 1,120(2.6) | 1,555(4.6) | <0.001 |
| Cardiac death | 75(0.2) | 80(0.2) | 0.046 |
| Nonfatal myocardial infarction | 241(0.6) | 224(0.7) | 0.049 |
| Nonfatal stroke | 1,213(2.8) | 1,286(3.8) | <0.001 |
|  |  |  |  |
| Secondary outcome | 3,861(8.9) | 3,913(11.7) | <0.001 |
| All cause death | 1,120(2.6) | 1,555(4.6) | <0.001 |
| Nonfatal myocardial infarction | 241(0.6) | 224(0.7) | 0.049 |
| Revascularization | 436(1.0) | 293(0.9) | 0.057 |
| Admission from heart failure | 189(0.4) | 198(0.6) | 0.003 |
| Ischemic stroke | 1,054(2.4) | 1,107(3.3) | <0.001 |

Values are presented as n (%).

ARB = angiotensin receptor blocker

CCB = calcium channel blocker

**Supplementary Table 8.** Incidence of clinical events in the previously diagnosed hypertensive group during a 3-year follow-up

| Variable | The primary therapy group | | p-value |
| --- | --- | --- | --- |
|  | ARB  (n=157,459) | CCB  (n=230,679) |  |
| Primary outcome (MACEs) | 8,127(5.2) | 16,550(7.2) | <0.001 |
| All cause death | 3,370(2.1) | 8,351(3.6) | <0.001 |
| Cardiac death | 234(0.1) | 586(0.3) | <0.001 |
| Nonfatal myocardial infarction | 798(0.5) | 1,347(0.6) | 0.001 |
| Nonfatal stroke | 4,536(2.9) | 8,161(3.5) | <0.001 |
|  |  |  |  |
| Secondary outcome | 13,773(8.7) | 25,226(10.9) | <0.001 |
| All cause death | 3,370(2.1) | 8,351(3.6) | <0.001 |
| Nonfatal myocardial infarction | 798(0.5) | 1,347(0.6) | 0.001 |
| Revascularization | 1,185(0.8) | 1,734(0.8) | 0.975 |
| Admission from heart failure | 758(0.5) | 1,530(0.7) | <0.001 |
| Ischemic stroke | 3,981(2.5) | 7,176(3.1) | <0.001 |

Values are presented as n (%).

ARB = angiotensin receptor blocker

CCB = calcium channel blocker

**Supplementary Table 9.** Incidence of clinical events under age 55 during a 3-year follow-up

| Variable | Under age 55 | | p-value |
| --- | --- | --- | --- |
|  | ARB  (n=74,976) | CCB  (n=55,962) |  |
| Primary outcome (MACEs) | 1,324(1.8) | 1,247(2.2) | <0.001 |
| All cause death | 351(0.5) | 360(0.6) | <0.001 |
| Cardiac death | 20(0.0) | 16(0.0) | 0.836 |
| Nonfatal myocardial infarction | 222(0.3) | 206(0.4) | 0.024 |
| Nonfatal stroke | 795(1.1) | 713(1.3) | <0.001 |
|  |  |  |  |
| Secondary outcome | 3,064(4.1) | 2,427(4.3) | 0.025 |
| All cause death | 351(0.5) | 360(0.6) | <0.001 |
| Nonfatal myocardial infarction | 222(0.3) | 206(0.4) | 0.024 |
| Revascularization | 318(0.4) | 230(0.4) | 0.716 |
| Admission from heart failure | 98(0.1) | 79(0.1) | 0.610 |
| Ischemic stroke | 606(0.8) | 514(0.9) | 0.032 |

Values are presented as n (%).

ARB = angiotensin receptor blocker

CCB = calcium channel blocker

**Supplementary Table 10.** Incidence of clinical events over age 55 during a 3-year follow-up

| Variable | Age 55 or older | | p-value |
| --- | --- | --- | --- |
|  | ARB  (n=125,752)) | CCB  (n=208,258) |  |
| Primary outcome (MACEs) | 9,202(7.3) | 18,116(8.7) | <0.001 |
| All cause death | 4,139(3.3) | 9,546(4.6) | <0.001 |
| Cardiac death | 289(0.2) | 650(0.3) | <0.001 |
| Nonfatal myocardial infarction | 817(0.6) | 1,365(0.7) | 0.842 |
| Nonfatal stroke | 4,954(3.9) | 8,734(4.2) | <0.001 |
|  |  |  |  |
| Secondary outcome | 14,570(11.6) | 26,712(12.8) | <0.001 |
| All cause death | 4,139(3.3) | 9,546(4.6) | <0.001 |
| Nonfatal myocardial infarction | 817(0.6) | 1,365(0.7) | 0.842 |
| Revascularization | 1,303(1.0) | 1,797(0.9) | <0.001 |
| Admission from heart failure | 849(0.7) | 1,649(0.8) | <0.001 |
| Ischemic stroke | 4,429(3.5) | 7,769(3.7) | 0.002 |

Values are presented as n (%).

ARB = angiotensin receptor blocker

CCB = calcium channel blocker

**Supplementary Table 11.** Predictors for MACEs in the newly diagnosed hypertensive group during a 3-year follow-up

| Risk Factor | Univariate analysis | | | | Multivariate analysis | | | |
| --- | --- | --- | --- | --- | --- | --- | --- | --- |
|  | HR | 95% CI | | p-value | HR | 95% CI | | p-value |
|  |  | Lower | Upper |  |  | Lower | Upper |  |
| ARB | 0.642 | 0.608 | 0.678 | <0.001 | 0.818 | 0.768 | 0.871 | <0.001 |
| Age |  |  |  |  |  |  |  |  |
| <40 | 1 |  |  |  | 1 |  |  |  |
| 40-49 | 1.524 | 1.188 | 1.956 | 0.001 | 1.460 | 1.092 | 1.950 | 0.011 |
| 50-59 | 2.546 | 2.012 | 3.223 | <0.001 | 2.433 | 1.852 | 3.196 | <0.001 |
| 60-69 | 4.348 | 3.440 | 5.495 | <0.001 | 4.062 | 3.098 | 5.328 | <0.001 |
| 70- | 13.498 | 10.732 | 16.976 | <0.001 | 12.712 | 9.731 | 16.606 | <0.001 |
| Female | 0.888 | 0.841 | 0.938 | <0.001 | 0.636 | 0.598 | 0.677 | <0.001 |
| Income |  |  |  |  |  |  |  |  |
| <25% | 1 |  |  |  | 1 |  |  |  |
| 25%-75% | 0.971 | 0.892 | 1.058 | 0.505 | 1.063 | 0.972 | 1.163 | 0.180 |
| >75% | 1.070 | 0.983 | 1.165 | 0.116 | 0.922 | 0.843 | 1.008 | 0.073 |
| Diabetes mellitus | 1.273 | 1.186 | 1.366 | <0.001 | 0.987 | 0.910 | 1.072 | 0.760 |
| Chronic kidney disease | 1.822 | 1.387 | 2.395 | <0.001 | 1.215 | 0.902 | 1.637 | 0.199 |
| Comorbidity channel index |  |  |  |  |  |  |  |  |
| 0 | 1 |  |  |  | 1 |  |  |  |
| 1 | 1.807 | 1.688 | 1.934 | <0.001 | 1.491 | 1.385 | 1.604 | <0.001 |
| 2 | 2.660 | 2.448 | 2.891 | <0.001 | 1.942 | 1.771 | 2.128 | <0.001 |
| >3 | 4.640 | 4.236 | 5.083 | <0.001 | 2.905 | 2.624 | 3.216 | <0.001 |

ARB = angiotensin receptor blocker

**Supplementary Table 12.** Predictors for MACEs in the previously diagnosed hypertensive group during a 3-year follow-up

| Risk Factor | Univariate analysis | | | | Multivariate analysis | | | |
| --- | --- | --- | --- | --- | --- | --- | --- | --- |
|  | HR | 95% CI | | p-value | HR | 95% CI | | p-value |
|  |  | Lower | Upper |  |  | Lower | Upper |  |
| ARB | 0.721 | 0.702 | 0.741 | <0.001 | 0.940 | 0.913 | 0.967 | <0.001 |
| Age |  |  |  |  |  |  |  |  |
| <40 | 1 |  |  |  | 1 |  |  |  |
| 40-49 | 1.613 | 1.248 | 2.086 | <0.001 | 1.713 | 1.298 | 2.259 | <0.001 |
| 50-59 | 2.456 | 1.917 | 3.147 | <0.001 | 2.658 | 2.035 | 3.473 | <0.001 |
| 60-69 | 4.561 | 3.564 | 5.836 | <0.001 | 4.773 | 3.657 | 6.228 | <0.001 |
| 70- | 14.322 | 11.205 | 18.307 | <0.001 | 15.063 | 11.552 | 19.640 | <0.001 |
| Female | 0.932 | 0.909 | 0.956 | <0.001 | 0.725 | 0.706 | 0.745 | <0.001 |
| Income |  |  |  |  |  |  |  |  |
| <25% | 1 |  |  |  | 1 |  |  |  |
| 25%-75% | 0.907 | 0.872 | 0.943 | <0.001 | 0.970 | 0.933 | 1.009 | 0.127 |
| >75% | 1.038 | 1.000 | 1.078 | 0.052 | 0.890 | 0.857 | 0.925 | <0.001 |
| Diabetes mellitus | 1.409 | 1.363 | 1.457 | <0.001 | 1.083 | 1.044 | 1.124 | <0.001 |
| Chronic kidney disease | 1.663 | 1.383 | 1.998 | <0.001 | 1.225 | 1.004 | 1.495 | 0.045 |
| Comorbidity channel index |  |  |  |  |  |  |  |  |
| 0 | 1 |  |  |  | 1 |  |  |  |
| 1 | 1.491 | 1.446 | 1.537 | <0.001 | 1.308 | 1.266 | 1.352 | <0.001 |
| 2 | 2.064 | 1.991 | 2.140 | <0.001 | 1.625 | 1.563 | 1.690 | <0.001 |
| >3 | 3.379 | 3.249 | 3.515 | <0.001 | 2.366 | 2.266 | 2.471 | <0.001 |

ARB = angiotensin receptor blocker

**Supplementary Table 13.** Predictors for MACEs under age 55 during a 3-year follow-up

| Risk Factor | Univariate analysis | | | | Multivariate analysis | | | |
| --- | --- | --- | --- | --- | --- | --- | --- | --- |
|  | HR | 95% CI | | p-value | HR | 95% CI | | p-value |
|  |  | Lower | Upper |  |  | Lower | Upper |  |
| ARB | 0.802 | 0.742 | 0.866 | <0.001 | 0.763 | 0.701 | 0.831 | <0.001 |
| Female | 0.634 | 0.584 | 0.688 | <0.001 | 0.618 | 0.565 | 0.675 | <0.001 |
| Income |  |  |  |  |  |  |  |  |
| <25% | 1 |  |  |  | 1 |  |  |  |
| 25%-75% | 0.973 | 0.869 | 1.089 | 0.632 | 0.942 | 0.839 | 1.058 | 0.316 |
| >75% | 0.716 | 0.636 | 0.807 | <0.001 | 0.668 | 0.590 | 0.756 | <0.001 |
| Diabetes mellitus | 1.594 | 1.435 | 1.771 | <0.001 | 1.250 | 1.106 | 1.414 | <0.001 |
| Chronic kidney disease | 1.422 | 0.883 | 2.291 | 0.148 | 0.925 | 0.533 | 1.605 | 0.781 |
| Comorbidity channel index |  |  |  |  |  |  |  |  |
| 0 | 1 |  |  |  | 1 |  |  |  |
| 1 | 1.428 | 1.304 | 1.564 | <0.001 | 1.344 | 1.218 | 1.482 | <0.001 |
| 2 | 1.926 | 1.705 | 2.175 | <0.001 | 1.782 | 1.558 | 2.038 | <0.001 |
| >3 | 3.580 | 3.082 | 4.150 | <0.001 | 3.077 | 2.597 | 3.659 | <0.001 |

ARB = angiotensin receptor blocker

CCB = calcium channel blocker

**Supplementary Table 14.** Predictors for MACEs not less than age 55 during a 3-year follow-up

| Risk Factor | Univariate analysis | | | | Multivariate analysis | | | |
| --- | --- | --- | --- | --- | --- | --- | --- | --- |
|  | HR | 95% CI | | p-value | HR | 95% CI | | p-value |
|  |  | Lower | Upper |  |  | Lower | Upper |  |
| ARB | 0.858 | 0.837 | 0.880 | <0.001 | 0.812 | 0.790 | 0.834 | <0.001 |
| Female | 0.815 | 0.796 | 0.835 | <0.001 | 0.800 | 0.780 | 0.821 | <0.001 |
| Income |  |  |  |  |  |  |  |  |
| <25% | 1 |  |  |  | 1 |  |  |  |
| 25%-75% | 0.949 | 0.914 | 0.985 | 0.006 | 0.942 | 0.907 | 0.978 | 0.002 |
| >75% | 1.065 | 1.027 | 1.104 | 0.001 | 1.047 | 1.010 | 1.086 | 0.013 |
| Diabetes mellitus | 1.310 | 1.269 | 1.352 | <0.001 | 1.110 | 1.072 | 1.150 | <0.001 |
| Chronic kidney disease | 2.130 | 1.813 | 2.502 | <0.001 | 1.418 | 1.192 | 1.686 | <0.001 |
| Comorbidity channel index |  |  |  |  |  |  |  |  |
| 0 | 1 |  |  |  | 1 |  |  |  |
| 1 | 1.399 | 1.359 | 1.441 | <0.001 | 1.379 | 1.337 | 1.424 | <0.001 |
| 2 | 1.842 | 1.780 | 1.907 | <0.001 | 1.797 | 1.732 | 1.865 | <0.001 |
| >3 | 2.870 | 2.764 | 2.979 | <0.001 | 2.783 | 2.672 | 2.899 | <0.001 |

ARB = angiotensin receptor blocker

CCB = calcium channel blocker
