# Supplementary figures and images for: Clinical outcomes between calcium channel blockers and angiotensin receptor blockers in hypertensive patients without established cardiovascular diseases during a 3-year follow-up
Source: Sci Rep. 2021 Jan 19;11:1783. doi: 10.1038/s41598-021-81373-7 (PMC7815918; doi:10.1038/s41598-021-81373-7)

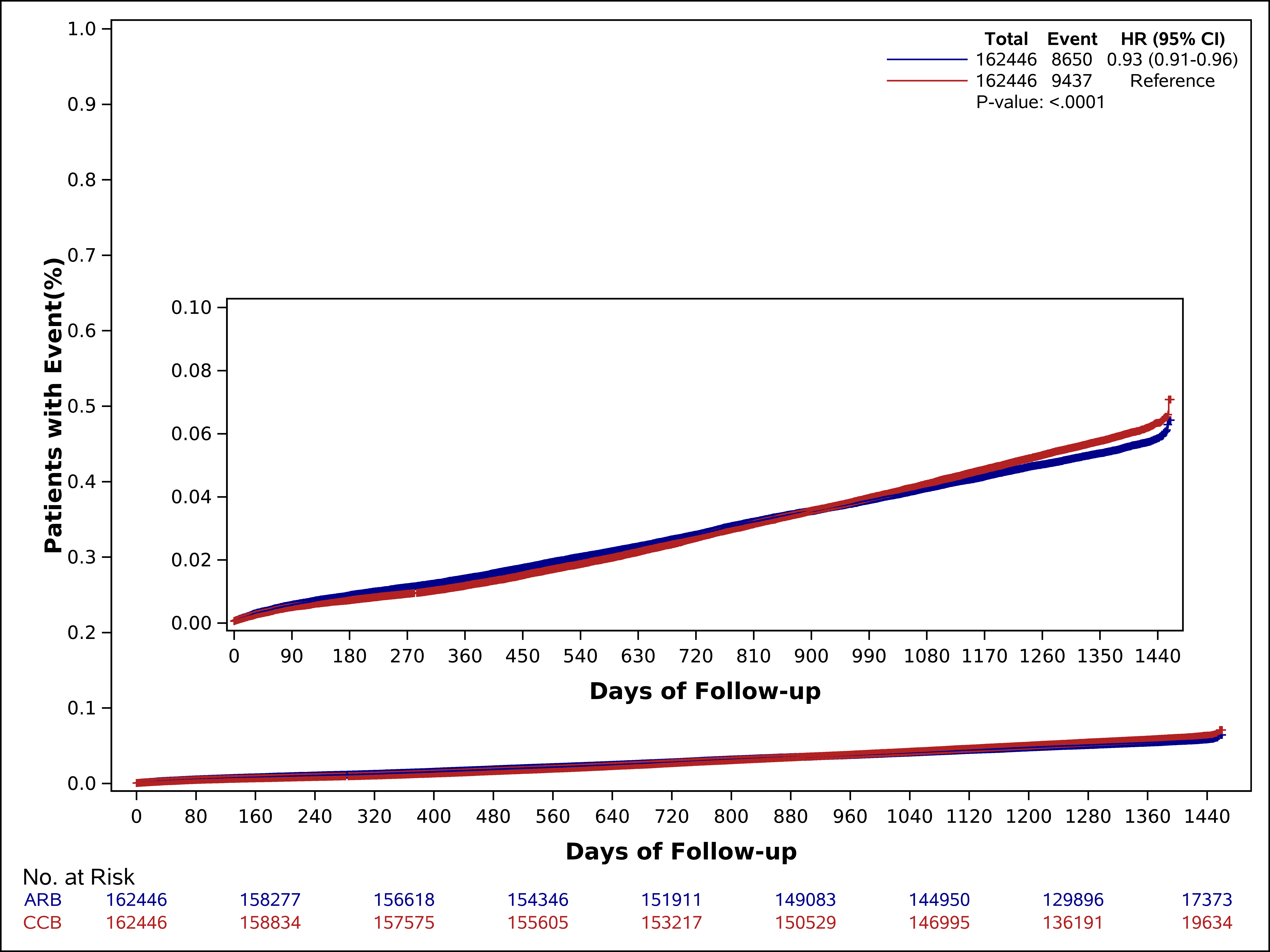

Supplement: Supplementary file 3 — Supplementary Information 3. [file 41598_2021_81373_MOESM3_ESM.jpg]

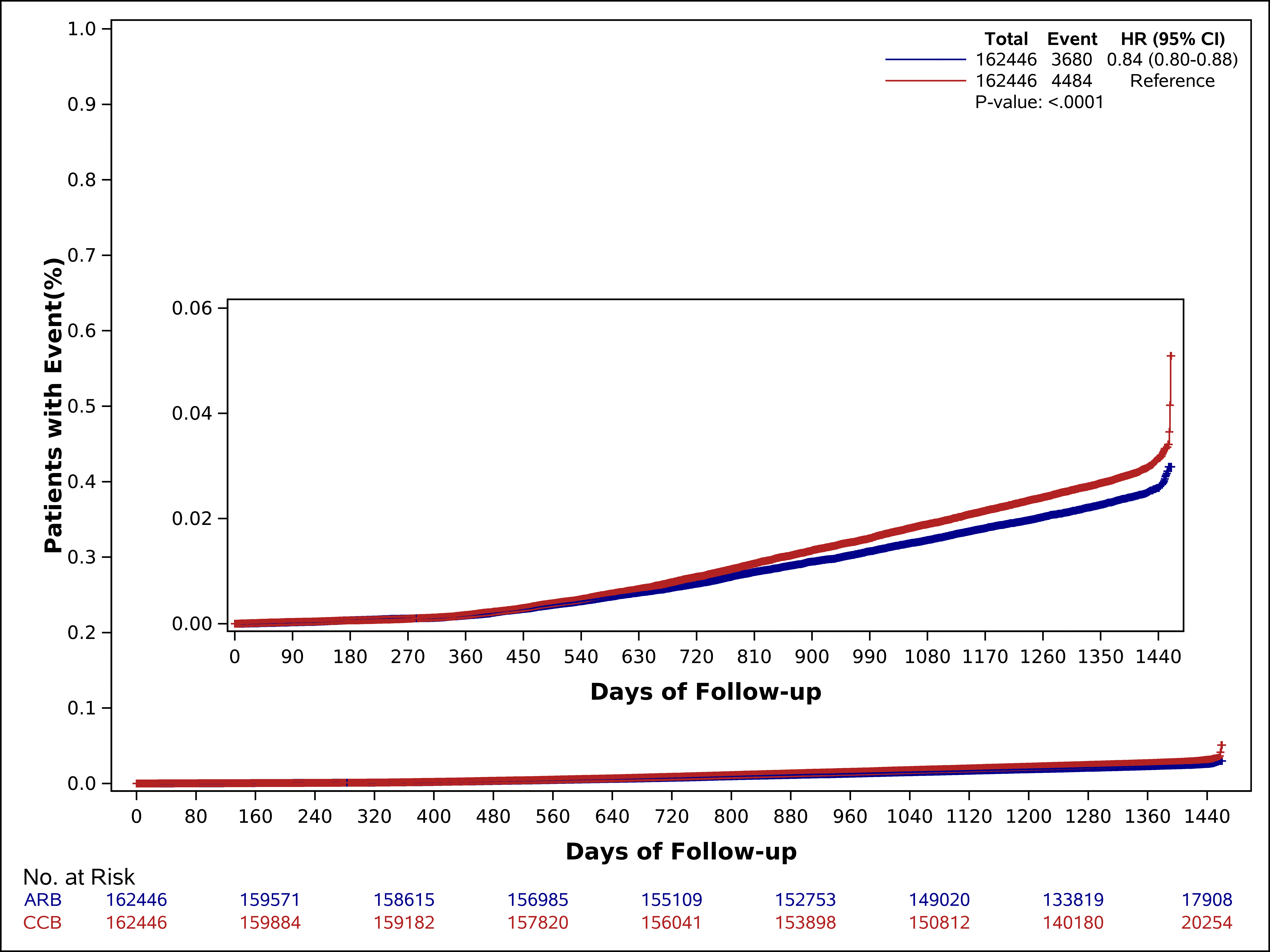

Supplement: Supplementary file 4 — Supplementary Information 4. [file 41598_2021_81373_MOESM4_ESM.jpg]

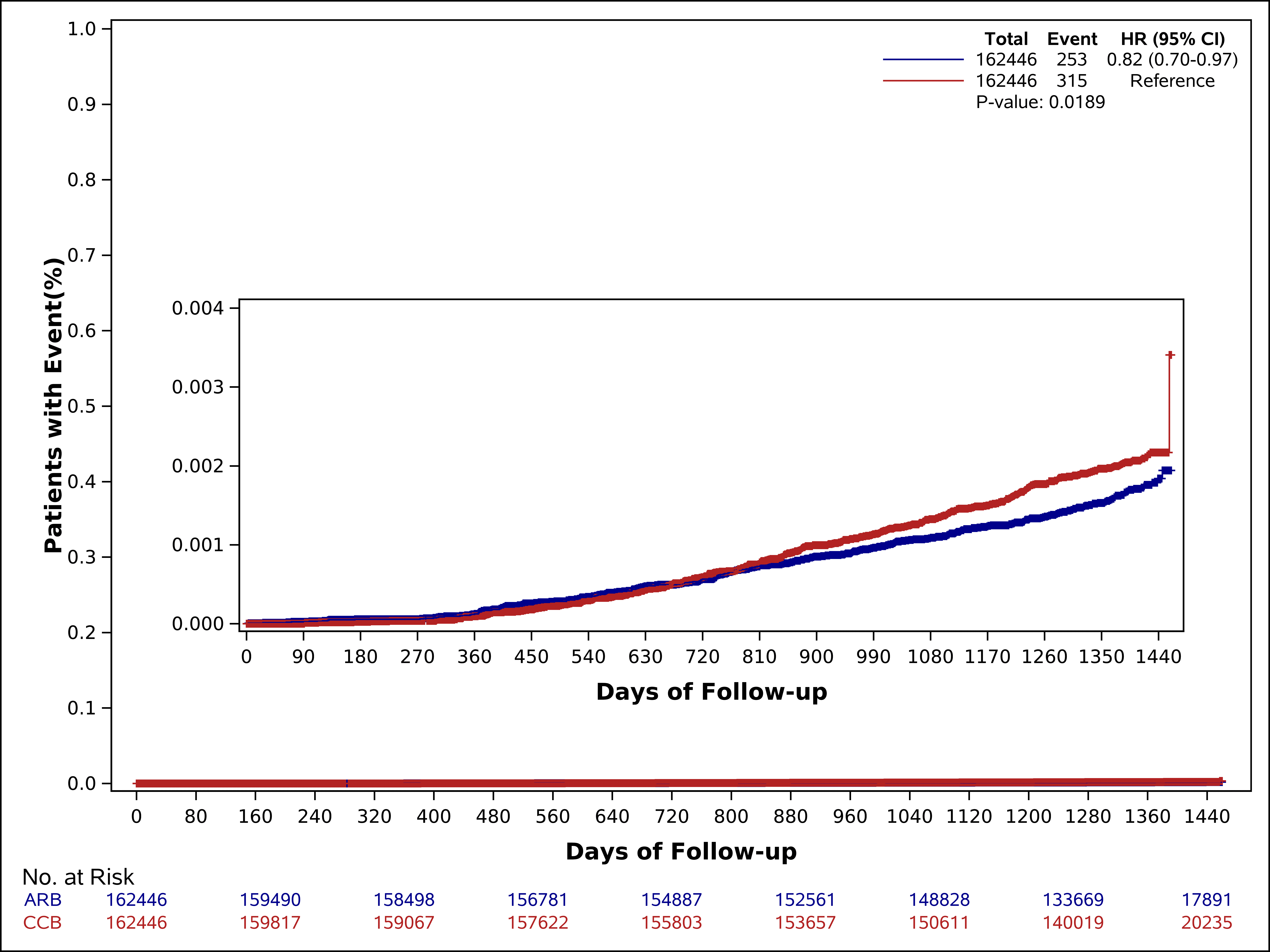

Supplement: Supplementary file 5 — Supplementary Information 5. [file 41598_2021_81373_MOESM5_ESM.jpg]

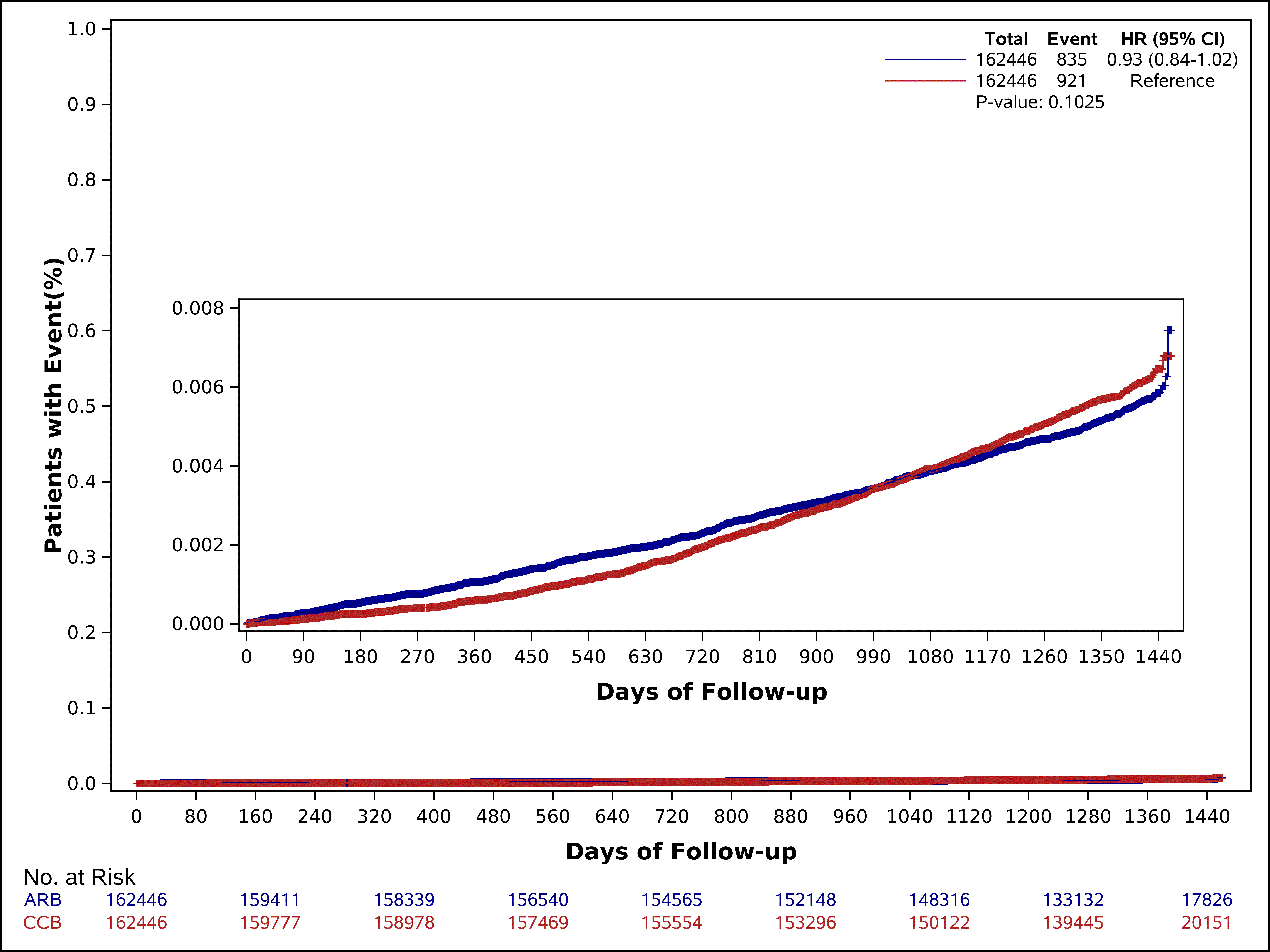

Supplement: Supplementary file 6 — Supplementary Information 6. [file 41598_2021_81373_MOESM6_ESM.jpg]

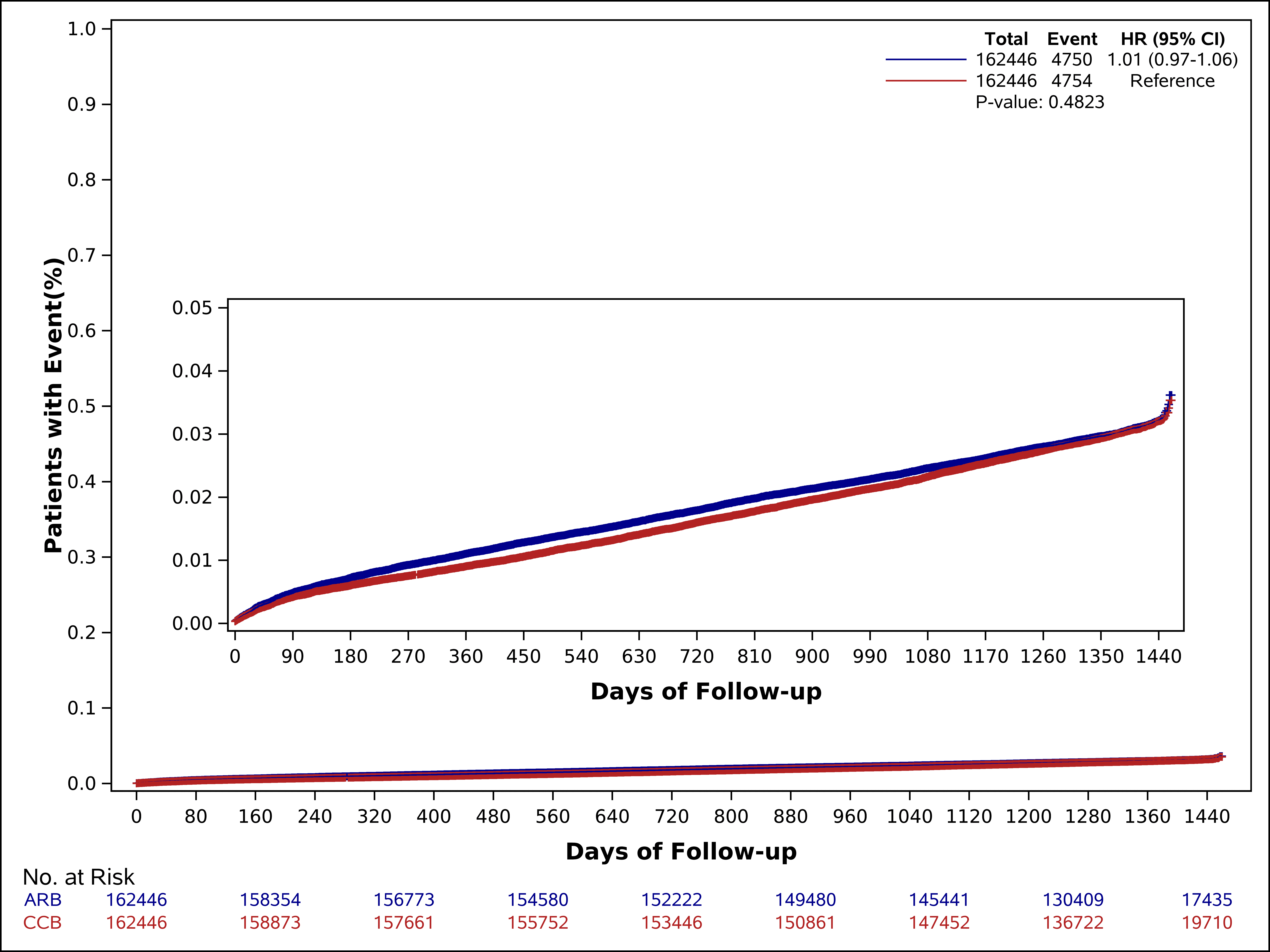

Supplement: Supplementary file 7 — Supplementary Information 7. [file 41598_2021_81373_MOESM7_ESM.jpg]
